# Cerebral Glucose Metabolism and Potential Effects on Endoplasmic Reticulum Stress in Stroke

**DOI:** 10.14336/AD.2022.0905

**Published:** 2023-04-01

**Authors:** Sichao Guo, Alexandra Wehbe, Shabber Syed, Melissa Wills, Longfei Guan, Shuyu Lv, Fengwu Li, Xiaokun Geng, Yuchuan Ding

**Affiliations:** ^1^China-America Institute of Neuroscience, Beijing Luhe Hospital, Capital Medical University, China; ^2^Department of Neurosurgery, Wayne State University School of Medicine, USA; ^3^Department of Neurology, Beijing Luhe Hospital, Capital Medical University, China; ^4^Harvard T.H. Chan School of Public Health, USA

**Keywords:** gluconeogenesis, glycolysis, stroke, endoplasmic reticulum stress

## Abstract

Ischemic stroke is an extremely common pathology with strikingly high morbidity and mortality rates. The endoplasmic reticulum (ER) is the primary organelle responsible for conducting protein synthesis and trafficking as well as preserving intracellular Ca2^+^ homeostasis. Mounting evidence shows that ER stress contributes to stroke pathophysiology. Moreover, insufficient circulation to the brain after stroke causes suppression of ATP production. Glucose metabolism disorder is an important pathological process after stroke. Here, we discuss the relationship between ER stress and stroke and treatment and intervention of ER stress after stroke. We also discuss the role of glucose metabolism, particularly glycolysis and gluconeogenesis, post-stroke. Based on recent studies, we speculate about the potential relationship and crosstalk between glucose metabolism and ER stress. In conclusion, we describe ER stress, glycolysis, and gluconeogenesis in the context of stroke and explore how the interplay between ER stress and glucose metabolism contributes to the pathophysiology of stroke.

## 1. Introduction

Stroke has a strikingly high mortality rate of 5.5 million worldwide each year, ranked as the second leading cause of death globally [1-3]. Various molecular processes, such as endoplasmic reticulum (ER) stress, are involved in the immediate and long-term physiological reactions to cerebral ischemia [4]. In the setting of ischemic stroke, normal oxidative phosphorylation is inhibited, and ATP production and glucose metabolism are interrupted. Anaerobic processes like glycolysis emerge as primary sources of energy in ischemic stroke, which increases lactate generation [5]. Recent research has demonstrated gluconeogenesis also becomes a key method of energy metabolism after stroke [6]. Here, we address the development of ER stress and changes in glucose metabolism (including glycolysis and gluconeogenesis) after stroke and contribute towards the discussion on stroke treatment.

## 2. ER Stress and Stroke

The ER is a eukaryotic cellular organelle within the cytoplasm that creates and modifies proteins, including folding and assembly when proteins are translocated to the ER lumen. Certain triggers, such as hypoxia and ischemia, disrupt ER homeostasis. Reduced blood flow due to arterial occlusion can lead to tissue hypoxia, a condition that quickly induces misfolding of proteins and ER stress. When blood flow is restored, reperfusion of affected tissues results in oxidative stress as well as altered ER redox status, which disrupts protein disulfide formation resulting in misfolding of ER proteins. The disruption of ER homeostasis leads to a signal induction pathway to maintain cell survival [7, 8].

**Figure 1.**
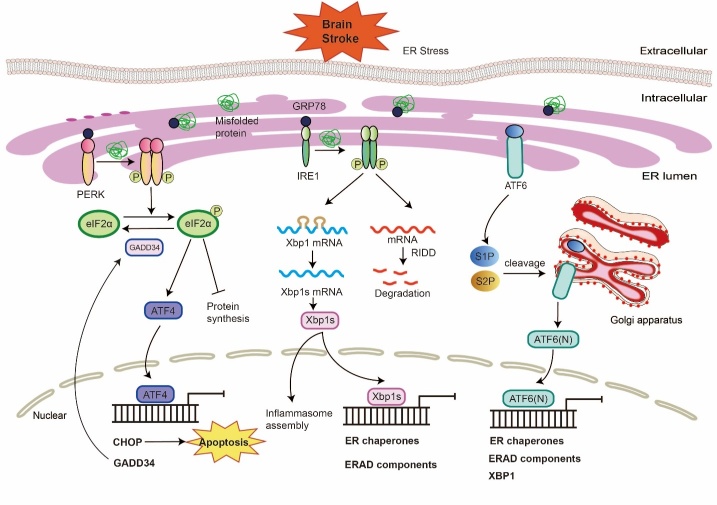
The UPR-ER stress response associated with cerebral ischemia mediated by IRE1, PERK, and ATF6 pathways. In ischemic conditions, ER stress activates UPR through induction of GRP78 dissociation from sensors proteins. *PERK pathway:* PERK activation is caused by ER stress through oligomerization and autophosphorylation in the serine/threonine kinase domain. By phosphorylating eIF2α, activated PERK leads to ER stress relief by tempering protein translation. The phosphorylated eIF2α, however, selectively enhances ATF4 translation to activated CHOP and GADD34 transcription. Following alleviation of ER stress, eIF2α is dephosphorylated by GADD34 pathways, resulting in protein translation restoration. However, CHOP induces cellular apoptosis if alleviation of ER stress does not occur. *IRE1 pathway:* Through oligomerization and autophosphorylation, IRE1 is initiated and results in increased RNase activity. Activated IRE1 prompts splicing of a 26-nucleotide-long intron in XBP1, followed by translation of the newly modified mRNA into active XBP1s transcription factor. XBP1s upregulates ER chaperone expression and levels of ERAD components, among other genes. Furthermore, IRE1-induced RIDD machinery alleviates ER stress through mRNA encoding ER-targeted proteins degradation, causing a lowered protein load into the ER. Active XBP1s promotes NLRP3-ASC/Caspase-1 inflammasome assembly in the regulation of neuronal pyroptosis. *ATF6 pathway:* Prompted by ER stress, ATF6 is translocated out of the ER and into the Golgi apparatus. Here, S1P and S2P proteolytically cleave ATF6 to expose the N-terminal region of ATF6, now termed ATF6(N). As an actived transcription factor, ATF6(N) upregulates genes necessary for production of ER chaperones, ERAD components, and XBP1.

Increased protein folding demand or disrupted protein folding upsets the equilibrium between protein folding demand and ER functional capacity. This imbalance causes misfolded proteins to accumulate within the ER lumen; this phenomenon is called ER stress [9]. Cells under mild ER stress separate from danger signals to offset ischemic injury via activation of survival favoring mechanisms, whereas pro-apoptotic mechanisms are favored with chronic ER stress, and act to damage nerve cells [4]. To prevent this, eukaryotic cells activate the unfolded protein response (UPR), a method to alter gene transcription and translation to respond to protein-folding flaws resulting from stressors [9, 10]. The UPR prevents cells from succumbing to stressors, however, prolonged UPR activation acts as an apoptotic signal for cells [11]. To determine a cell’s future, the UPR functions as a stress sensor while also regulating downstream transcription factors; these factors monitor misfolded and unfolded protein aggregation and help recode genes [12, 13]. In the context of ischemic stroke, cessation of oxygen and glucose delivery to neurologic tissue triggers the ER stress cascade. Low oxygen and glucose disrupt proper ER functioning with respect to protein folding/processing. Investigation of protein expression from these complex UPR pathways provides insight on how ischemic stroke causes brain damage.

The UPR begins once protein kinase RNA-like ER kinase (PERK), inositol-requiring protein 1 (IRE1) as well as activating transcription factor 6 (ATF6), are activated. In the balanced state of neuronal homeostasis, these three bind to glucose regulated protein 78 (GRP78)/binding immunoglobulin protein (Bip), an ER chaperone, due to their devitalized state. Unbound GRP78 binds hydrophobic sections of polypeptides to facilitate proper folding in ischemic stroke and initiates the UPR [4, 14, 15]. After a new GRP78 protein is created to bind the unfolded proteins, ATF6, PERK, and IRE1 are inactivated, restoring ER function and cessating the UPR response [16]. UPR alleviates ER stress by upregulating cofactors, chaperones, and enzymes found in the ER while downregulating protein translation, resulting in neuroprotection during ischemic stroke. However, a prolonged UPR due to longstanding, high levels of ER stress paradoxically programs cell death instead of the conventional protective effect on cells during cerebral ischemia stroke (Fig. 1)[4, 17].

### 2.1. PERK pathway

PERK is a kinase; it creates oligomers and, as a reaction to ER stress, autophosphorylates to become active. Once PERK is activated, it phosphorylates eukaryotic initiation factor 2α (eIF2α), eIF2α then rearranges the ribosome to attenuate protein translation [18]. This PERK-eIF2α pathway decreases ER unfolded protein load through temporarily inhibiting protein synthesis [18]. In contrast, phosphorylation of eIF2α also selectively promotes gene translation on genes containing an upstream open reading frame (uORF), including activating transcription factor 4 (ATF4) [19, 20]. When there is ER stress, ATF4 activates transcription of certain UPR target genes, particularly CCAAT/enhancer binding protein (C/EBP) homologous protein (CHOP), a transcription factor involved in apoptosis as well as growth arrest and DNA damage-inducible protein 34 (GADD34) [17, 21]. Once ER stress resolves, eIF2α is dephosphorylated from GADD34’s interaction with type 1 protein serine/threonine phosphatase (PP1). This functions to re-establish proper protein translation. If ER stress remains, however, CHOP induces apoptosis of cells [17, 19, 22, 23].

During stroke, the PERK signaling pathway is activated. PERK-specific knockout mice have been found to have bigger infarct volumes and worse neurologic function scores with low levels of p-eIF2a expression post-middle cerebral artery occlusion (MCAO) compared to control mice [24]. The underlying mechanism may be that neurons are protected from ischemic stress due to PERK-induced eIF2α phosphorylation with resulting translation suppression and avoiding unfolded protein load [24]. Investigators observed that the peri-infarct area of rodents with cerebral ischemia had upregulated GADD34, a protein phosphatase that targets eIF2α, in its microglia and neurons. To reverse ischemic damage, dephosphorylation of eIF2α by GADD34 is crucial to restart protein translation, but will simultaneously further incite damage through misfolded protein accumulation [4, 25, 26]. However, ischemia/reperfusion (I/R) also promotes the initiation of the PERK/ATF4/CHOP-related apoptotic pathway including increasing expression of eIF2α, p-eIF2α, PERK, p-PERK, ATF4, CHOP, caspase 12, and cleaved-caspase 3 factors. Studies have shown that inhibition of PERK-CHOP-mediated apoptosis is protective against cerebral I/R [27]. Endoplasmic reticulum metalloprotease 1 (ERMP1), a zinc-binding protease, may be the upstream factor of the GRP78-PERK-CHOP signaling pathway. miR-9 targets ERMP1 to inhibit ER stress to protect against ischemic insult [7]. Canopy FGF signaling regulator 2 (CNPY2) is a member of the canopy family and found in various rat tissues. CNPY2 expression in the ischemic penumbra was found to be greater post-stroke and expression of ER stress apoptotic proteins (CHOP and caspase-3) was also significantly increased in the ischemic penumbra [28]. This may be due to the CNPY2 binding partner being switched from GRP78 to PERK, then selectively activating the PERK-CHOP-mediated apoptosis signaling pathway [28]. Through C1q/TNF-related protein1 (CTRP1), cells are protected from cerebral I/R through reduced ER stress and apoptosis. This occurs through CTRP1 inhibition of PERK signaling pathways [27]. Hes1 (hairy and enhancer of split 1), a transcription factor that serves many roles within the nervous system, was recently correlated with the UPR and ER stress-triggered apoptosis. Hes1 functions to modulate PERK/eIF2α/ATF4/CHOP signaling pathways and serves to protect against I/R injury. Knockdown of Hes1 increased infarction sizes and worsened neurological outcomes and increased apoptosis [29].

### 2.2. IRE1 pathway

IRE1α is widely distributed in a variety of tissues in reaction to ER stress and encompasses two domains including a serine/threonine kinase domain and an endoribonuclease domain [30]. GRP78 detaches from IRE1α and facilitates oligomerization and auto-phosphorylation of IRE1[17]. Then, UPR activation results in IRE1-dependent splicing of a 26-nucleotide intron from the X box binding protein 1 (XBP1) mRNA, promoting its translation into active transcription factor spliced XBP1 (XBP1s) protein [31, 32]. This XBP1 protein increases the UPR targeting of gene expression, specifically ER chaperones, including GRP78 and ER-associated protein degradation (ERAD) components[17, 33]. The RNase domain of IRE1 causes multiple RNA cleavages in a pathway called regulated IRE1-depedent decay (RIDD)[34]. This mechanism alleviates ER stress through degradation of mRNAs that code for ER-target proteins, reducing protein load into the ER [17].

Stroke induces the activation of IRE1 and the processing of XBP1 mRNA. Then the ER chaperones including GRP78 protein are synthesized in areas with ischemic neuronal damage to restore ER function [31, 35]. XBP1 deletion following both transient and permanent MCAO resulted in worsened outcomes [36]. Notably, O-linked β-N-acetylglucosamine modification of proteins, a process called O-GlcNAcylation, is an XBP1-dependent process found to be activated in young mice neurons in the penumbra. This process was markedly impaired in older mice [36]. This research suggests a protective role of the IRE1/XBP1 pathway after stroke. However, cerebral I/R facilitates unspliced XBP1 (XBP1u) mRNA splicing to form the active version of XBP1s. Active XBP1s promotes nucleotide-binding domain (NOD)-like receptor protein 3 (NLRP3)/ASC/Caspase-1 assembly of inflammasome, which subsequently creates inflammatory cytokines during regulation of pyroptosis [37]. Microglia under oxygen-glucose deprivation/reoxygenation (OGD/R) conditions exhibited increased levels of interleukin-1 β (IL-1 β), interleukin-6 (IL-6) and tumor necrosis factor-α (TNF-α); this condition and these cytokines cause increased XBP1 and p-IRE1α expression in addition to neuronal apoptosis. Notably, administration of Icariin (a flavonol glycoside being investigated for ischemic stroke therapy) reversed the levels of these factors. However, overexpression of IRE1 increased ER stress-mediated apoptosis while diminishing Icariin’s protective role [38].

### 2.3. ATF6 Pathway

ATF6 is also a transmembrane transcription factor which activates genes for ER response. This factor contains an N-terminal b-ZIP domain in cellular cytoplasm. In response to unfolded protein accumulation in the ER, ATF6 unbinds from GRP78 and translocates to the Golgi apparatus. There, 90KD molecular weight ATF6 is activated by site-1 protease (S1P) and site-2 protease (S2P) into 50 kDa ATF (N) active form. The active cytoplasmic region of ATF6 (N), which contains the N-terminal region, then migrates to the nucleus to regulate gene expression [4, 17, 39]. Once in the nucleus, ATF6 demonstrates both pro-apoptotic and pro-survival mechanisms of cell control [4]. ATF6 acts simultaneously with other transcription factors to activate ER chaperone expression, Ca2^+^ pumps, antioxidant genes, and ERAD genes. Responding to ER stress, ATF6 triggers production of XBP1 mRNA. IRE1 then splices this XBP1 mRNA. ATF6 and XBP1 are noted to occasionally co-activate specific genes [33, 40, 41].

In the post-stoke setting, elevated autophagic activity levels during early reperfusion time are hypothesized to add to the neuroprotection provided by ATF6, making the ATF6 UPR pathway imperative to stroke outcomes [42]. When the ATF6 gene is deleted, this results in greater infarct volumes and neuronal death in the periinfarct region post-MCAO in mice. These phenotypes in ATF6^-^/^-^ mice were associated with lower activation of astroglia, reduced glial scar formation, and greater tissue damage expansion into non-infarct areas. ER stress in the basal ATF6^-^/^-^ astrocytes shut down signal transducer and activator of transcription 3 (STAT3)-glial fibrillary acidic protein (GFAP) signaling, a key astroglial activation pathway [43]. By increasing GRP78 expression and antioxidant enzyme catalase, ATF6 activated pharmacologically has demonstrated neuro-protection in myocardial, renal, and cerebral models of I/R [44]. On the contrary, ATF6 also contributes to activation of the ER stress apoptosis pathway [45, 46].

Combined, these three pathways constitute UPR’s adaptive response to ER stress through protein synthesis inhibition, increased chaperone protein expression resulting in increased folding capacity, and increased breakdown of irreversibly misfolded proteins. In ischemic stroke, inadequate oxygen and glucose delivery to the brain causes ER stress. This subsequently activates the three pathways involved in the UPR response. Investigating expression levels of proteins involved in these pathways provides insight on how ischemic stroke causes brain damage. Studying elements of the ER stress pathway that are activated in response to ischemic insult can help identify therapeutic targets for stroke management.

## 3. Drugs and Treatments Reduce Damage Caused by Stroke-Associated with ER Stress

Certain drugs and treatments can reduce the damage caused by stroke by targeting different ER stress pathways (Table 1).

**Table 1:** Drugs and treatments reduce damage caused by stroke associated with ER stress.

Drugs/treatments	Related protein changes	Effects
Targeting PERK pathway		
tPA	Decreasing PERK/CHOP pathway activity through binding to GRP78.	tPA prevents overactivation of ER stress, resulting in neuron protection in a thromboembolic model of stroke.
Parecoxib (COX-2 inhibitor)	Suppressing CHOP and Foxo1 nuclear translocation, increasing GRP78 and ORP150 levels.	Parecoxib protects neurons from apoptosis provoked by cerebral I/R injury via its ability to interfere with ER cell stress pathways.
Nafamostat mesylate (NM)	Decreasing expression of GRP78/CHOP/p-eIF2α.	NM exerted neuroprotective effects after focal cerebral I/R injury in rodents by inhibiting ER stress, demonstrating by infarct size reduction and neurological deficit improvement.
3-bromo-7-nitroindazole (3-BNI)	Decreasing expression of GRP78/CHOP.	3-Bromo-7-nitroindazole inhibited the cerebral infarct size, volume of edema, and increased restoration of neurologic function in diabetic stroke.
Apelin-13 (casein kinase 2 (CK2) activator)	Decreasing GRP78/p-eIF2α/ATF4/CHOP levels through upregulating CK2 expression.	Apelin-13 attenuated ER stress-mediated the cerebral infarct and neuronal apoptosis in ischemic stroke.
Ketogenic diets (KD) or β-hydroxybutyrate (BHB)	Decreasing GRP78/p-PERK/p-eIF2α/ATF4/CHOP expression.	KD or BHB attenuates recruitment of Drp1 and ER stress, reducing inflammation and reducing ischemic injury.
Melatonin	Decreasing p-PERK/p-eIF2α/ATF4/CHOP expression.	Melatonin decreased infarct volumes and individual cortical lesion sizes while also increasing the amount of surviving neurons through attenuating post-ischemic ER stress.
Endovascular mesenchymal stem cells (MSCs)	Reducing p-GRP78/p-eIF2α/ATF4/CHOP levels.	Administering MSCs after stroke improves functional neurologic outcomes, reduces infarct sizes, increases survival of neurons, and normalizes biochemical parameters through modulating ER stress-mediated apoptosis through the BDNF/TrkB signaling pathway.
Sodium 4-phenylbutyrate (SPB)	Attenuation in upregulation GRP78/CHOP.	SPB ameliorated cerebral infarct size and edema volume in ischemic strokes associated with type 2 diabetes by reducing ER stress.
4-Phenylbutyric Acid (4-PBA) and Lithium	Downregulation p-PERK/p-eIF2α expression and upregulation Akt activity.	4-PBA and lithium work cooperatively to promote cell survival and reduce apoptotic damage induced by OGD.
Postconditioning	Increasing GRP78 expression and decreasing CHOP/caspase-12/p-Akt expression.	Cerebral ischemic postconditioning confers neuroprotection from I/R injury through suppressing apoptosis mediated by ER stress.
Remote ischemic postconditioning (RIPostC)	Increasing GRP78 expression, decreasing p-eIF2α/caspase-12/CHOP expression.	RIPostC provides neuroprotection from I/R injury in rodents through decreasing apoptosis caused by ER stress.
Astragalin (AST)	Decreasing expression of GRP78/CHOP/BAX/cleaved caspase-3/caspase-12. Increasing expression of Bcl-2.	AST protect against I/R induced brain injury, attenuated the expression levels of the ER stress related protein, as well as its downstream apoptotic mediators.
Ginsenoside Rg1 (Rg1)	Decreasing expression of p-PERK/p-eIF2??/CHOP/ATF4/cleaved caspase-12/cleaved caspase-3/BAX. Increased Bcl-2 expression.	Rg1 modifies stress-responsive genes, which helps prevent neuronal ER stress induced by glutamate via the PERK-eIF2α-ATF4 signaling pathway.
Targeting IRE1 pathway		
Icariin (ICA)	Decreasing expression of p-IRE1/XBP1/NLRP3/caspase-1/IL-1β/IL-6/TNF-α.	ICA’s anti-inflammatory properties could aide in ischemic stroke treatment through inhibiting the IRE1/XBP1s pathway.
Targeting ATF6 pathway		
Liquiritin (LQ)	Decreasing expression of Keap1/GRP78/ATF6. Increasing Nrf2/ZO-1/Claudin-5 expression.	LQ protects cerebral microvascular endothelial cells from I/R injury in humans, which may be related to its inhibitory effect on both ER and oxidative stress, maintains the integrity of BBB.
Targeting multiple pathways		
Granulocyte-colony stimulating factor (G-CSF)	Decreasing expression of GRP78/cleaved -ATF6/XBP1/ATF4/eIF2α/CHOP.	G-CSF confers neuroprotection to damaged neurons through suppression of ER stress against ischemic global stroke.
Cilostazol	Decreasing expression of GRP78/ATF6/p-IRE1/p-PERK.	Cilostazol prevented I/R induced disruption of tight junctions of brain endothelial cells via ER stress inhibition.
Combined therapy with S-M ethyl-N,N-diethylthiolcarbamate sulfoxide (DETC-MeSO) and taurine	Decreasing expression of GRP78/cleaved ATF6/ATF4/p-IRE1/CHOP.	The combination therapy reduced infarct size, post-stroke neurological deficits, ameliorating the damage from cerebral ischemia by attenuating the ER stress pathway.
Sc-222227 (Protein tyrosine phosphatase 1B (PTP1B) inhibitor)	Decreasing expression of cleaved ATF6/p-IRE1/p-PERK/p-eIF2α.	sc-222227 reduced ER stress, microglial activation, and autophagy while promoting M2 polarization, resulting reduced neuronal damage and neurodeficits.
γ-glutamylcysteine (γ-GC)	Decreasing GRP78/p-IRE1/p-PERK/p-eIF2α/CHOP expression.	γ-glutamylcysteine (γ-GC) attenuated apoptosis and brain injury by inhibiting the ER stress pathway in ischemic brain and OGD/R cells.
Erythropoietin (EPO)	Suppressing the expression of GRP78/ATF6α/CHOP/Foxo1/ATF4.	Low dose EPO treatment provided neuroprotection after acute ischemic stroke through inhibition of the ER stress response in neuronal tissue and isolated microvessels.
Sodium nitrite (SN)	Decreasing expression of p-PERK/ATF6/CHOP.	SN treatment increased cell viability and conferred neuroprotection by reducing ROS-mediated ER stress resulting from OGD insult.
Exercise postconditioning (PostE)	Decreasing GRP78/IRE1/PERK/ATF6/CHOP/caspase-12/BAX expression and increasing Bcl-2/SIRT1 expression.	Exercise regimens completed post stroke decrease cerebral edema and infarct volume, neurological deficits, production of ROS, and apoptosis by inhibiting ER stress.
Xuefuzhuyu decoction (XS)	Decreasing PERK/XBP1/ATF6/CHOP/BAX/MMP-9 expression and increasing GRP78/TIMP-1/Bcl-2 expression.	XS may impede neuronal apoptosis through suppressing signaling of apoptosis dependent on ER-stress and blood-brain barrier protection.
YiQiFuMai (YQFM) powder	Decreasing GRP78/CHOP/ATF6/ATF4/p-eIF2??/XBP1/cleaved caspase-3/caspase-12 expression.	YQFM reduces ischemic injury severity via modifying signaling pathways related to ER stress.
Longxuetongluo Capsule (LTC)	Decreasing expression of GRP78/p-IRE1/p-eIF2??/p-PERK/CHOP/TPAF2/cleaved PARP/cleaved caspase-3/cleaved caspase-9/p- JNK/p-Erk1/2/p-P38/BAX.	LTC provides neuroprotection from I/R injury via MAPK and ER stress-related mechanisms.
Forsythiaside A (FA)	Decreasing expression of p-PERK/p-IRE1/CHOP/caspase-3/caspase-9. Increasing Bcl-2/Nrf2/Nqo1/GST expression.	FA lessened ischemic damage by modifying the activation of Nrf2 and ER stress pathways.
Vitamin B and rapamycin co-treatment	Decreasing expression of GRP78/p-IRE1/p-eIF2??/CHOP/ATF4/ATF6.	Vitamin B and rapamycin co-treatment may help relieve ischemic damage caused by reversing cellular autophagy disturbances and ER caused by HHcy.

### 3.1. Targeting PERK pathway

Drugs such as tissue-type plasminogen activator (tPA) have been found to have pharmacological effects that affect ER stress. tPA has shown neuroprotective effects by decreasing PERK activity through binding to GRP78, leading to lower levels of apoptotic factors like CHOP. This ultimately prevents overactivation of ER stress, resulting in neuron protection [11]. Parecoxib, a COX-2 inhibitor used as an anti-inflammatory and analgesic agent, also protects neurons from apoptosis due to cerebral I/R injury. In the ischemic penumbra, parecoxib demonstrated suppression of CHOP, forkhead box protein O 1 (Foxo1), and caspase-12 nuclear translocation. This also caused an increase in endoplasmic reticulum chaperones GRP78 and oxygen-regulated protein 150 (ORP150) expression, both of which are associated with neuroprotection and reduced cell stress. These findings indicate that the neuro-protection conferred by parecoxib may be due to its ability to interfere with ER cell stress pathways [47, 48]. Nafamostat mesylate (NM), a serine protease inhibitor, was found to exert neuroprotective effects post-focal cerebral I/R insult in rats. NM reduced the MCAO-mediated increase in CHOP and GRP78, suggesting that its neuroprotection stems from its CHOP and GRP78 activity inhibition [49]. Diabetes mellitus is a common comorbidity and major risk factor in ischemic stroke. Hyperglycemia induces nitric oxide toxicity following I/R, enhancing brain ischemia. It has also been speculated that nitric oxide could promot cell death through CHOP in the ER stress pathway. Consequently, 3-bromo-7-nitroindazole (3-BNI), a selective and potent neuronal nitric oxide synthase (nNOS) inhibitor, was found to act against ER stress and type 2 diabetes mellitus-associated focal cerebral I/R injury through DNA fragmentation reduction and decrease of both GRP78 and CHOP [50]. Casein kinase 2 (CK2) is a protein that is part of the ER stress and apoptotic pathways through eIF2-ATF4-CHOP cascade regulation. Increased levels of CK2 facilitate cell survival, while lower levels promote apoptosis [51]. Cerebral I/R injury resulted in activated eIF2-ATF4-CHOP signaling and decreased CK2 expression, further promoting neuronal apoptosis. Apelin-13 was found to significantly upregulate expression of CK2 and inhibit activation of eIF2-ATF4-CHOP through CK2 signaling, thus reducing rodent infarct size and neuronal apoptosis [52]. Recent studies suggest that dietary intake may impact neural responses to ischemic damage. Ketogenic diets (KD) increase serum concentrations of ketone bodies, like acetoacetate and β-hydroxybutyrate (BHB), that function as glucose alternatives for cerebral metabolism. Studies assessing ketone bodies have found that high levels of ketone bodies reduce cerebral edema and infarct size after stroke and have the ability to prevent neuronal death from glucose deprivation or hypoxia [53]. After MCAO, ketone bodies suppress ER stress and dynamin-related protein 1 (Drp1) mitochondrial translocation, inhibiting ER stress activation of NLRP3 inflammasome and protecting mitochondrial integrity. These findings indicate that increased serum levels of ketone bodies, by means such as a ketogenic diet, may be protective against ER stress-mediated damage after stroke [54, 55]. Melatonin, an endogenously produced hormone from the pineal gland that regulates circadian rhythm, has also been investigated as a method to protect the ischemic brain from pathologic ER stress. Melatonin treatment at reperfusion onset after MCAO demonstrated decreased volumes of infarction and cortical lesion sizes along with increased survival of neurons. Melatonin significantly modulated protein levels by decreasing levels of ER stress-associated expression of proteins like p-PERK, p-eIF2α, ATF4, and CHOP in a dose-dependent fashion in the ischemic core and penumbra [56, 57]. Quality of life and neuroprotection were found to be increased when mesenchymal stem cells were utilized post-stroke. This was demonstrated by ER stress-mediated apoptosis modulation through the brain-derived neurotrophic factor/tropomyosin receptor kinase B (BDNF/TrkB) signaling pathway [58]. Sodium 4-phenylbutyrate (SPB) is a chemical chaperone in ER stress that is also of interest in type 2 diabetes mellitus patients, as it has also resulted in reduced insulin resistance and dysfunction of beta cells in rats and humans. Treatment with SPB significantly reduced brain I/R damage, demonstrated through decreased cerebral infarct and edema volumes. SPB significantly reduced DNA fragmentation while also significantly attenuating upregulation of GRP78, CHOP and activation of caspase-12 [59, 60]. 4-phenylbutyric acid (4-PBA), an ammonia scavenging fatty acid, also behaves as a chaperone to decrease misfolded proteins reserve inside the ER, thereby diminishing ER stress. Furthermore, combined lithium and 4-PBA treatment rescued the previously ER stress-suppressed Akt biosynthesis pathway, known to modulate neurotransmission, synaptic plasticity, neurogenesis, and apoptosis. This indicates that 4-PBA and lithium synergistically promote cell survival and reduce apoptotic damage induced by ischemia [61]. The ER stress pathophysiologic pathways were also examined in the context of ischemic postconditioning, a well-described clinical technique for attenuating ischemic damage after a cerebrovascular event. Cerebral ischemic post-conditioning decreased brain I/R injury, resulting in infarct size reduction, decreased cell apoptosis, and phosphatidylinositol-3kinase/Akt (PI3K/Akt) pathway-mediated alterations in proteins involved in ER stress-mediated apoptosis. Indeed, postconditioning reduced CHOP expression and caspase-12 activation, as well as increased the expression of GRP78, a protective molecular chaperone in this pathway [62]. Similarly, remote ischemic postconditioning (RIPostC) was found to increase expression of protective GRP78 and Bcl-2 while decreasing expression of phosphorylated-eIF2α, caspase-12, cleaved-caspase-3, Bim, and CHOP. This further shows RIPostC’s ability to decrease ER stress response-induced apoptosis[63, 64]. Astragalin (AST) has been found to be neuroprotective following I/R injury through suppressing GRP78, CHOP, and caspase-12 [65]. Ginsenoside Rg1 (Rg1), a key component in Panax notoginseng saponins, causes inhibition ER stress-induced apoptosis in MCAO-induced rats through inhibition of the PERK-ATF4-CHOP pathway [66].

### 3.2. Targeting IRE1 pathway

Icariin (ICA), a flavonol glycoside derived from epimedium brevicornum maxim (Berberidaceae), has been postulated to decrease IL-1β, IL-6 and TNF-α expression through IRE1/XBP1s pathway inhibition [67].

### 3.3. Targeting ATF6 pathway

Derived from the plant Glycyrrhiza uralensis, Liquiritin (LQ) is a flavonoid Fisch that promotes proliferation, migration, and angiogenesis of cells while reducing apoptosis. This is thought to be due to its inhibitory effect on both ER by targeting GRP78/ATF6 and oxidative stress. Furthermore, LQ maintains blood brain barrier (BBB) integrity following hypoxia/reoxygenation [68].

### 3.4. Targeting multiple pathways

Granulocyte-colony stimulating factor (G-CSF) is an FDA approved drug that reduces neuronal degeneration while also contributing to long-term plasticity post-ischemia. Through suppressing ER and mitochondrial stress along with maintaining cellular homeostasis via decreased apoptosis, G-CSF confers neuroprotection to damaged neurons [45, 69]. Cilostazol, a neuroprotective PDE3 inhibitor, has been found to lessen I/R induced disruption of tight junctions of brain endothelial cells via ER stress inhibition [70]. An Nmethyl-D-aspartate (NMDA) receptor partial antagonist, S-M ethyl-N,N-diethylthiolcarbamate sulfoxide (DETC-MeSO), which is an FDA-approved treatment for alcohol dependence disorder, was also investigated for its ability to ameliorate damage from cerebral ischemia by attenuating the ER stress pathway. Combination therapy with DETC-MeSO and taurine reduced ER stress-mediated apoptosis by inhibiting the ATF6, IRE-1, and PERK pathways [71]. Investigations have found increased microglial protein tyrosine phosphatase 1B (PTP1B) expression, indicative of harmful microglial activation resulting from cerebral I/R. PTP1B is a regulator of numerous central nervous system (CNS) processes and exacerbates neuro-inflammation. Its inhibitor, sc-222227 was found to reduce ER stress, microglial activation, and autophagy while promoting M2 polarization. Sc-222227 was also found to reduce IR-induced neuronal damage and neurological deficits [72]. Endogenous gluthathione is heavily consumed after cerebral ischemic episodes and its precursor, γ-glutamylcysteine (γ-GC), was found to attenuate apoptosis and brain injury resulting from ischemia and reperfusion. Further studies showed γ-GC lessened penumbra neuronal apoptosis through inhibition of ischemia-induced ER stress pathway components PERK and IRE1α [73]. Erythropoietin (EPO), an endogenous hormone that increases red blood cell production, was also evaluated as an exogenous stroke treatment in the context of ER stress. Administering low-dose EPO at the beginning of reperfusion post-MCAO suppressed I/R-induced upregulation of the GRP78-ATF4 -CHOP sequence and that of caspase-3 in cerebral micro-vessels and brain tissue. It also reduced levels of Foxo1 and ATF6α, which have similar roles in ER stress [74]. Low-dose sodium nitrite (SN) provides a partial neuroprotective effect via reduced reactive oxygen species (ROS) ER stress instigation following OGD insult [75]. Exercise postconditioning (PostE) could induce neuroprotection by regulating SIRT1 in the ROS/ER stress pathway [76, 77]. The neuroprotective effects of some natural ingredients after ischemia are related to ER stress. Ligusticum chuanxiong (CX), and Radix Paeoniae Rubra (CS) are herbs traditionally used in Chinese medicine for their analagesic, anti-hemostatic, and anti-inflammatory properties. The combination of these two therapies, known as Xuefuzhuyu decoction (XS) was found to significantly reduce expression of ER stress-related factors PERK, XBP1, ATF6, and CHOP while increasing GRP78 expression. Of note, the effects were greatest when the therapies were combined, thus confirming a synergistic effect of CX and CS [78]. YiQiFuMai (YQFM) powder, another traditional Chinese medicine, is popularly used in cerebrovascular disease management. Cell incubation after MCAO and OGD-induced stress increased cell viability and inhibited apoptosis. This was demonstrated by its broad inhibition of ER stress pathways, including that of the GRP78/ATF6/ATF4 and caspase-12 cascades [79]. Longxuetongluo Capsule (LTC), a compound commonly used clinically to treat ischemic stroke in China, provides neuroprotective effects through ER stress and MAPK mechanisms following I/R injury [80]. A different compound, Forsythiaside A (FA), has also been postulated to decrease ischemic damage through nuclear factor-erythroid 2-related factor 2 (Nrf2) and ER stress activation mediation [81]. Vitamin B and rapamycin co-treatment may relieve ischemic damage by reversing cellular autophagy and ER stress defections caused by hyperhomocystinemia (HHcy) [82].

## 4. Cerebral Glucose Metabolism and Stroke

The neural environment must be preserved within a strict homeostatic range to preserve normal brain function [83]. Glucose is the brain's most important and almost exclusive metabolic fuel. The homeostatic of CNS is very susceptible to disruption in ischemic stroke. Ischemic stroke rapidly results in insufficient cerebral blood supply, causing severe glucose deprivation, consequently disturbing cellular homeostasis, and eventually resulting in neuronal death [84]. The CNS possesses various mechanisms to maintain optimal levels of glucose in the brain. After a stroke, ATP production and glucose metabolism homeostasis is disrupted, and glycolysis process becomes a major process for ATP production. Moreover, recent research has demonstrated that gluconeogenesis also plays an important role in cerebral energy supply after stroke.

### 4.1. Glycolysis

Higher rates of glucose metabolism relative to the rate of oxygen utilization, also termed hyperglycolysis, are elicited by reduced blood supply during ischemic stroke [85, 86]. The brain consumes large quantities of energy from aerobic metabolism under normal conditions.

**Figure 2.**
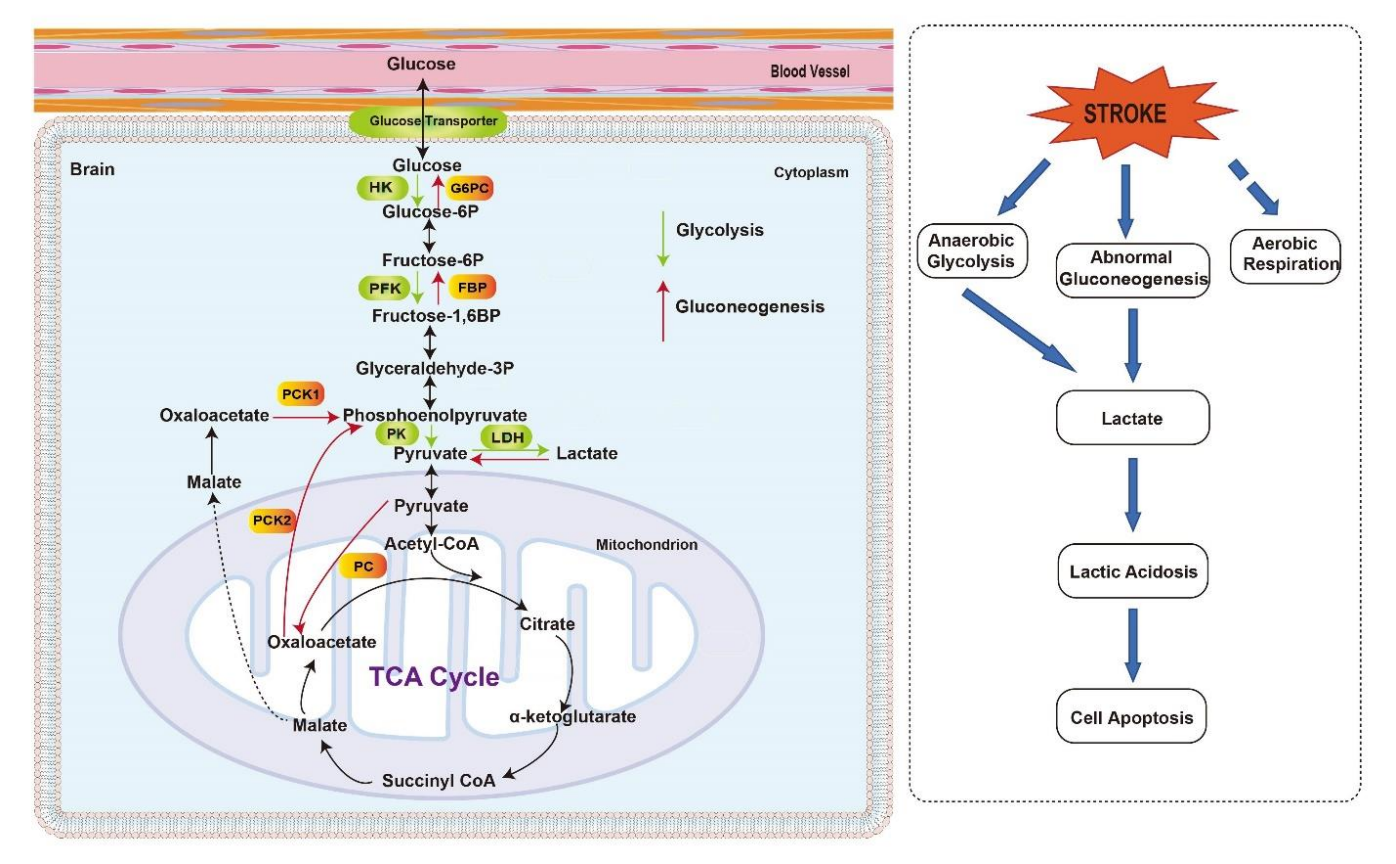
Glycolytic and gluconeogenesis pathways in the brain (left) and changes in glycolytic and gluconeogenesis after brain stroke (right). Gluconeogenesis and glycolysis share several of the same enzymes, which allow reversal of specific reactions in either process. However, there are some steps that are irreversible in the glycolysis and gluconeogenesis pathway that require different rate-limiting enzymes, e.g. Hexokinase (HK), Phosphofructokinase (PFK), Pyruvate kinase (PK) during glycolysis and Glucose-6-Phosphatase (G6PC), Pyruvate carboxylase (PC), Fructose 1,6-bisphosphatase (FBP), Phosphoenolpyruvate carboxykinase (PCK) during gluconeogenesis. During stroke, aerobic respiration is inhibited, leading to decreased ATP levels and blood perfusion to the brain while increasing anaerobic glycolysis and lactate accumulation. At the same time, abnormal brain gluconeogenesis can also exacerbate lactic acid accumulation. This accumulation of lactic acid can worsen neuronal apoptosis.

Changes in glucose metabolism have been recognized as a major pathogenesis in ischemic stroke. Following ischemic stroke, cerebral glucose and oxygen delivery are compromised, impairing oxidative phosphorylation. Consequently, the brain rapidly shifts its primary method of metabolism from oxidative phosphorylation to hyperglycolysis in order to meet the ischemic brain’s high energy demands. I/R injury models have shown increased glycolytic pathway activity within the ischemic penumbra reported for up to 24 hours after reperfusion began [87, 88]. Hyperglycolysis cannot meet the cells’ energy demands and resulting in lactic acidosis and ROS production post-stroke, contributing to brain damage [87-89]. Glucose transporter 1(GLUT-1) is the main channel for glucose transport in the body, with increased activity in astrocytes and endothelial cells, whereas neurons predominantly use glucose transporters 3 (GLUT-3) [90, 91]. Hypoxic and ischemic conditions are known to upregulate the expression of these two transporters[92]. Phosphofructokinase-1 (PFK-1) is the rate-limiting enzyme in glycolysis [93], which is stimulated by ATP breakdown products during I/R injury [94]. In hypoxic/ischemic conditions, Lactate dehydrogenase (LDH) generates lactate using pyruvate as a substrate, contributing to a metabolic acidotic state and neuronal death (Fig. 2) [95-97].

### 4.2. Gluconeogenesis

The biochemical changes that occur in ischemic stroke, such as upregulation of the glycolytic metabolic pathway, mediate inflammatory and oxidative damage in the CNS. Increased oxidative injury in the brain is a consequence of hyperglycolysis [98]. However, clinical trials that trials targeting hyperglycolysis have been ineffective [96], suggesting an incomplete understanding of cerebral glucose metabolism [6]. Cerebral gluconeogenesis may fill the “missing link” in the underlying pathophysiology of ROS injury and acidosis post-ischemic stroke [5, 6]. We still lack knowledge about gluconeogenesis, which in theory is the reverse metabolic pathway of glycolysis [99, 100] in the stroke brain.

In a recent stroke study using rats, it was found that hepatic gluconeogenesis is significantly upregulated [101]. On day one after cerebrovascular ischemia, rats had higher plasma concentrations of fasting blood glucose and insulin[101]. These same authors employed quantitative real-time PCR and found elevated gluconeogenic enzyme transcript levels (fructose 1,6-bisphosphatase (FBP), glucose-6-phosphatase (G6PC), and phosphor-enolpyruvate carboxykinase (PCK)) in the liver of rodents experiencing cerebrovascular ischemia compared to those undergoing the sham operation. This implies that hyperglycemia observed after cerebrovascular ischemia may be a consequence of increased transcription of hepatic gluconeogenic genes [101].

Gluconeogenesis is a metabolic process which changes non-carbohydrate carbon substrates, such as amino acids and triglycerides, to glucose [99]. Gluconeogenesis is one way mammals maintain blood glucose levels when there is insufficient supply, such as in states of hypoglycemia [6, 99]. While gluconeogenesis has conventionally been associated with peripheral metabolism[102], recent works suggest this process also happens in the brain[5]. Since gluconeogenesis is dependent on ATP, one theory is that cerebral gluconeogenesis may facilitate disturbances in glucose metabolism during I/R injury through aggravating acidosis and oxidative damage [5, 6, 103]. The isoforms of PCK, PCK-1 and PCK-2, are located in the cytoplasm and mitochondria, respectively. Both isoforms catalyze the irreversible conversion of oxaloacetate (OAA) into phosphoenolpyruvate (PEP) and carbon dioxide. Moreover, the function of PCK is independent of ATP [104]. Since mitochondrial oxidative phosphorylation is damaged by ischemic injury and anaerobic glycolysis does not generate enough ATP to preserve neuronal function, gluconeogenesis may be increased after ischemic injury in order to meet energy demands. However, during gluconeogenesis, several ascending gluconeogenic enzymes necessary for glucose production from OAA depend on ATP. Due to inadequate levels of ATP required for generation of glucose following ischemia, this gluconeogenesis pathway may not function properly, and instead may increase lactic acidosis and oxidative injury [5, 6]. A recent study has confirmed that rate-limiting gluconeogenic enzyme PCK is upregulated in the brain after ischemic insult. This upregulation was associated with reduced levels of substrate (OAA), higher levels of product (PEP and glucose), as well as higher levels of ROS and lactate (Fig. 2) [5, 6, 105].

Moreover, the high incidence of hyperglycemia post ischemic stroke could be partially attributed to preexisting aberrations in glucose metabolism. Abnormal gluconeogenesis may lead to hyperglycemia after stroke [106]. There is a higher incidence rate of cell death, larger infarct volume, brain edema, and hemorrhagic transformation in animals with hyperglycemia. Acute and subacute lactate production is associated with acute hyperglycemia. This is explained by increased anaerobic glycolysis from ischemic stroke-related depletion of ATP and insufficient blood perfusion. The oxidative phosphorylation process is disrupted. Oxygen is not delivered to tissues and resulting glycolysis produces lactic acid as a byproduct. This process is hypothesized to be driven by high intracellular glucose levels (Fig. 2)[96].

## 5. ER Stress and Glucose Metabolism

### 5.1. ER Stress and Glucose Metabolism in Other Pathological or Physiological Conditions

**Figure 3.**
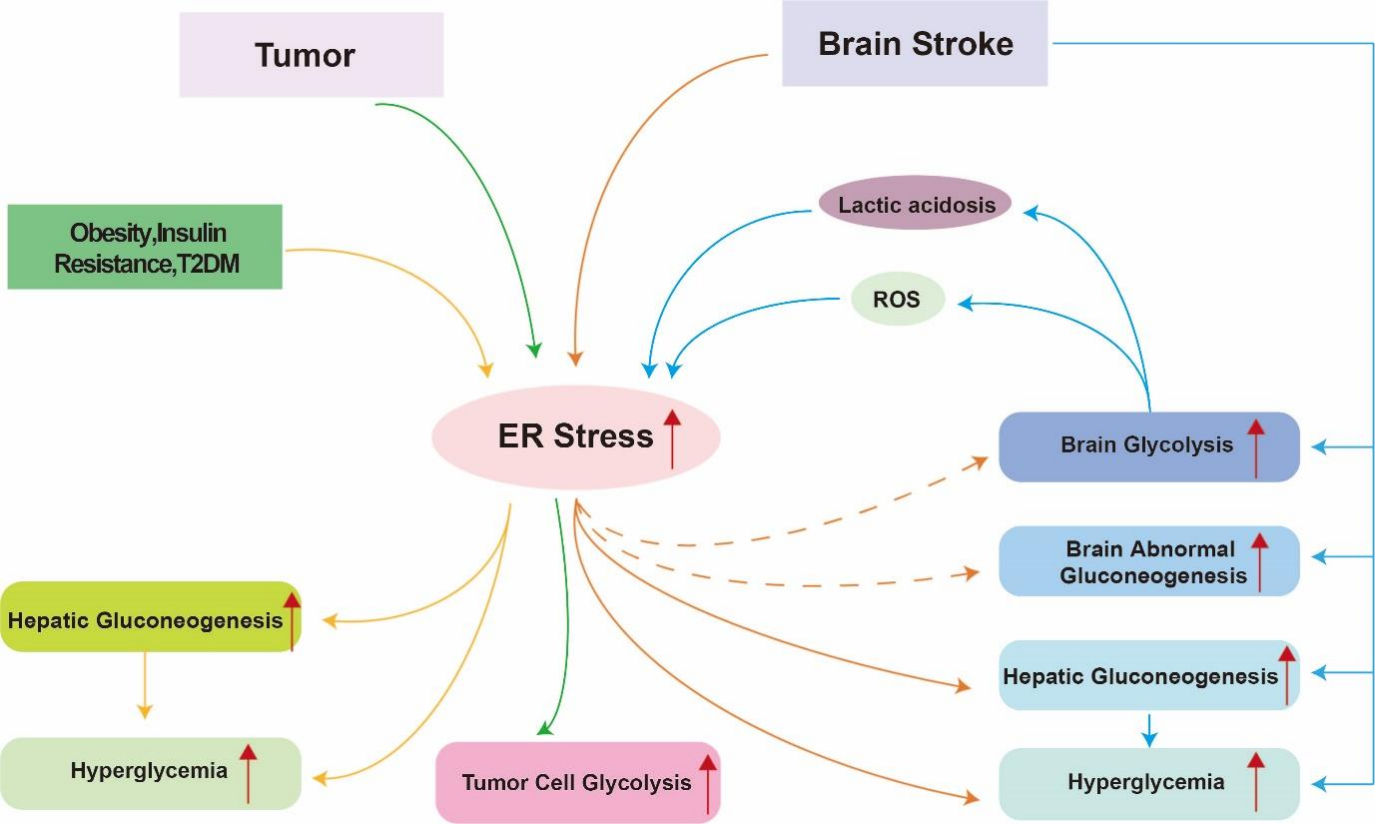
Relationship between ER stress and glucose metabolism in different disease models. In the liver, ER stress is tightly connected to type 2 diabetes mellitus, insulin resistance, and obesity. ER stress leads to increase gluconeogenesis in the liver in these diseases, which may further result in hyperglycemia. In tumor cells, ER stress leads to increased glycolysis. Moreover, in the brain, stroke can lead to increased anaerobic glycolysis as well as promote abnormal gluconeogenesis, resulting in neuronal damage. At the same time, stroke also promotes the process of gluconeogenesis in the liver, leading to hyperglycemia. In addition, ER stress can be activated during brain stroke. ROS and lactic acidosis may be two factors that indirectly link ER stress and glycolysis. How the glucose metabolism is affected through ER stress process after stroke is an important research direction. Existing studies mainly reported that ER stress promotes hepatic gluconeogenesis and hyperglycemia after stroke. Future research may focus on the specific relationship and crosstalk between ER stress and stroke-induced abnormalities in brain glucose metabolism, including glycolysis and gluconeogenesis.

As two important glucose metabolism processes, ER stress is related to glycolysis and gluconeogenesis in other pathological or physiological conditions as well, which will be briefly discussed below (Fig. 3).

Glycolysis is critical for metabolism in rapid cellular generation and proliferation, specifically in cancer cells [107]. For example, human glioma tissues showed significant activation of PERK. When silenced, there is a noted reduction of glycolysis and related Akt activation [108]. The ER stress facilitation of glycolysis is mediated by ATF4 in human lung carcinoma epithelial cells [109]. Upregulation of ER stress via the GRP78/p-PERK pathway leads to elevation of Nrf2 expression, which promotes glycolytic activity as well as antioxidant properties [110]. In Drosophilia models, S2 cells under ER stress exhibited upregulation of LDH and glycolytic genes due to ATF4, with downregulation of genes related to the citric acid cycle and respiratory chain complex [111]. Furthermore, IRE1 activity during hypoxic episodes increased levels of hypoxia-inducible factor-1alpha (HIF-1α) and GLUT-1 genes independent of XBP1 [112].

In the liver, ER stress is tightly associated with type 2 diabetes mellitus, insulin resistance, and obesity [113]. ER stress increases hepatic gluconeogenesis in these diseases. Studies have found that hepatic gluconeogenesis can be activated through AMP-activated protein kinase (AMPK)-heme oxygenase 1 (HO-1) activation of ER stress [114, 115]. Continuous ER stress in lean mice resulted in hyperglycemia via increased gluconeogenesis in the liver [116]. Further studies indicate that ER stress can inhibit IL-6/STAT3 pathways, through Janus kinase (JAK)2 dephosphorylation and STAT3 deacetylation, that decrease hepatic gluconeogenic enzymes [117]. Hypothalamic ER stress has been associated with increased hepatic gluconeogenesis in mice, as reported by Schneeberger et al. This results in disruption of glucose homeostasis in the liver, increasing chances of metabolic disorders[118]. ER stress also can suppress AMPK signaling while simultaneously causing the activation of PEPCK gene transcription via increased CCAAT/enhancer-binding protein (C/EBPβ) and phosphorylation of cAMP-responsive element binding (CREB) expression levels [119]. A decrease in the hepatic eIF2α signaling pathway leads to decreased gluconeogenic gene expression, resulting in reduced glucose production in the liver [120].

### 5.2. ER Stress and Glucose Metabolism in the Liver in Stroke

Glucose metabolism in the liver, especially gluconeogenesis, is relatively active, and glucose metabolism in the liver during stroke is related to ER stress. ER stress in the liver caused by obesity, a significant risk factor for stroke, has been found to increase insulin resistance and gluconeogenesis, ultimately leading to diabetes. Obesity notably increases stroke risk by several other mechanisms as well, including increased atherosclerosis and hypertension [121, 122]. Acute ischemic stroke can induce hyperglycemia even in patients without a diabetic history. Cerebral ischemia exposed rats, compared to control rats, demonstrated higher levels of glucose, hepatic gluconeogenic enzymes, insulin, and insulin resistance index. After acute cerebral ischemia, ER stress is activated in the liver. This, combined with impaired hepatic insulin signaling was found post-stroke [101, 123]. This study indicates that acute cerebral ischemia promotes ER stress-mediated liver gluconeogenesis, which incites the damaging sequelae of hyperglycemia (Fig. 3).

### 5.3. Indirect Relationship between Glucose Metabolism and ER stress

ER stress and glucose metabolism may also be indirectly linked through ROS production in stroke. A recent study has shown nicotinamide adenine dinucleotide phosphate oxidase (NOX) -derived ROS contributes to ER stress activation by oxidating proteins in hypertension. NOX1 and NOX4, chiefly found in the plasma membrane and ER respectively, take charge of increasing production of basal ROS. NOX1 is associated with irreversibly oxidating and phosphorylating the UPR sensor PERK, versus NOX4, which is part of a feedforward relationship with ER stress response as well as IRE1α oxidation [124]. Glucose metabolism, including glycolysis, may promote ROS production by activating the NOX pathway during I/R injury [92, 98]. After cerebral ischemia, excessive levels of ROS disturb and inhibit protein synthesis and incite DNA damage, resulting in brain damage [125]. Evolving evidence shows that ER stress can be triggered by excessive ROS in pathological states including stroke [73]. Studies suggest that the products of glycolysis may activate the ER stress pathway, impacting stroke outcomes (Fig. 3).

Lactic acidosis causes a decrease in intracellular pH, a serious consequence of hyperglycolysis after ischemia [96]. ER stress is also a known pivotal mediator of cell death in ischemia. There may be a link between lactic acidosis and ER stress [126]. Lactic acidosis causes greater expression of several UPR genes, such as ATF4, CHOP, XBP1 in cancer cells [127]. Similar results can also be found in human vascular endothelial cells (EC) [128] and astrocytes [126]. A vascular EC model showed that acidic extracellular pH activates the proton-sensing receptor G protein-coupled receptor 4 (GPR4) and stimulates all three ATF6, PERK, and IRE1 pathways [128]. Moreover, acidosis upregulates astrocyte GRP78 and caspase-12 expression and also leads to IRE1 dissociation from GRP78 after acidosis, inducing caspase-12 cleavage into its active form, leading to caspase-mediated cell death [126]. These results indicate that lactic acid, a product of anaerobic glycolysis, may activate ER stress in stroke (Fig. 3).

### 5.4. Other Potential Link between Glucose Metabolism and ER Stress in Stroke and Related Pathologies Condition

Diabetes is a significant risk factor for ischemic stroke that worsens brain damage, partially through ER stress aggrevating I/R injury. In I/R models, diabetic rats demonstrated greater infarct volumes and neurological deficits verus control rats, which is thought to be due to CHOP/GADD153 and caspase-12 activated cerebral ER stress and cell death [59]. The BBB, composed of brain capillary endothelial cells (BCEC), maintains glucose levels in the brain but is notably altered by cerebral ischemia. ER protein expression and glycolysis were markedly increased, indicating that hypoxia/reoxygenation of BCEC causes adaptive expression to increase glycolytic proteins, protein synthesis, and stress response proteins. This indicates that proteins involved in ER function, glycolysis, and cytoskeletal reorganization are heavily involved in cell adaptation and survival after ischemic insult [129]. Methylglyoxal (MGO), a glycolysis pathway byproduct, is the cause of hyperglycemia’s detrimental effects. The glyoxalase system functions to remove MGO, but this system is impaired in those with diabetes, and resulting in increased MGO concentration. ER stress is conjectured to impact atherosclerosis and vascular disease and may cause cell apoptosis if it persists. If these cells function to stabilize plaques, this increases the likelihood of stroke development through plaque disruption. MGO causes ER stress inside vascular smooth muscle cells, and with a failed glyoxalase system, leads to MGO-induced ER stress and resultant apoptosis, plaque rupture, and subsequent stroke [130]. However, ROS in ischemic stroke causes further damage to neurons and glial cells. Cellular glutathione in the pentose phosphate pathway (PPP) helps defend against the harmful capabilities of ROS. PPP activity and GSH levels were elevated in astroglia when exposed to acutely increased glucose concentrations, causing ROS production to decrease. Environments with chronically elevated glucose increased ER stress and PPP activity. Both acute and chronically elevated levels of glucose led to astroglial activation of the PPP and prevention of ROS production, indicating that rapid glucose reduction enhances ROS toxicity. The results suggest that rapid glucose concentration reduction could negatively impact the protective system of astroglia while possibly explaining why there is little evidence of the benefits of strict glucose control in the acute phase of stroke [131].

### 5.5. Influence of Aging and Gender

Aging and gender are two important factors affecting stroke prognosis. Aging promotes brain cell vulnerability and exacerbates cellular injury under pathological conditions [132, 133]. Aging is associated with unfolded and/or abnormally folded protein accumulation and the ER stress related UPR loses its ability to generate the adaptive response [134]. In ischemic conditions, aging impairs brain tissue recovery and worsens neurologic outcomes [135]. Aging can also destroy collateral circulation, disturbing brain reperfusion after ischemic stroke [136]. In aged rats, severe inhibition of tricarboxylic acid (TCA) cycle may be heightened compared to adult animals in ischemic conditions [137]. Moreover, in aged rats, creation of an anaerobic glucose metabolism end product is higher than during adulthood. This may play a role in tissue acidosis. As a consequence, the aging brain may not be as capable of meeting metabolic demands under emergent ischemic conditions [137]. However, the literature of how aging relates to cerebral glucose metabolism and ER stress in the setting of stroke is scant. The relationship of aging with ER stress and cerebral glucose metabolism in stroke should be further investigated in future studies.

There is mounting research focused on understanding gender differences in post-stroke outcomes. It has been reported that there is a higher incidence of stroke among women compared to men, as well as worse stroke outcomes. This may also be influenced by factors such as prestroke function, age, and incidence of atrial fibrillation by sex [138]. Treatment with estrogen has been found to be beneficial after stroke, however, these effects are age and sex dependent. Estrogen therapy in particular may be detrimental in older women [135, 139]. ER stress may also contribute to sex differences in ischemic disease. Sex differences in ANG II-induced brain ER stress have been found to contribute to sex differences in ANG II-mediated hypertension[140]. Studies have shown sex influenced type 1 diabetic-related brain metabolic changes. Anaerobic glycolysis was significantly altered, particularly within the striatum, midbrain, hypothalamus and hippocampus in male mice, but not in female mice [141]. However, adequate literature discussing how sex differences influence cerebral glucose metabolism and ER stress in the setting of stroke could not be found, and this may be a meaningful direction for future research.

## 6. Conclusions and Future Directions

ER stress plays an important role in the molecular processes underlying neurologic damage from ischemic stroke. Drugs and treatments targeting ER stress may improve the prognosis after stroke. Furthermore, glycolysis and gluconeogenesis, as important glucose metabolism processes, are of great significance to the homeostasis of energy metabolism after stroke. Moreover, ER stress may also be related to glycolysis and gluconeogenesis in tumor metabolism and hepatic gluconeogenesis. Some studies have discussed this complexity and illustrated the influences they exert on each other. These studies indicate that glucose metabolic pathways and associated dysfunctional states may be related to stroke and ER stress, which can exacerbate neuron dysfunction. Oxidative stress and lactic acidosis may indirectly link ER stress and glucose metabolism in stroke. However, the detailed relationship and mechanisms of specific glucose metabolism processes such as glycolysis, gluconeogenesis, and ER stress after ischemic stroke should be further investigated. We speculate there is a complex interplay between ER stress and glucose metabolism in the context of ischemic stroke. Future studies may focus on the specific relationship between ER stress and stroke-induced glucose metabolism abnormalities, serving as a promising target after stroke and provide novel avenues for post-stroke treatment.
